# The Genetic Etiology Diagnosis of Fetal Growth Restriction Using Single-Nucleotide Polymorphism-Based Chromosomal Microarray Analysis

**DOI:** 10.3389/fped.2021.743639

**Published:** 2021-10-15

**Authors:** Yu'e Chen, Yingjun Xie, Yuying Jiang, Qi Luo, Lijing Shi, Shuhong Zeng, Jianlong Zhuang, Guorong Lyu

**Affiliations:** ^1^Ultrasonography, Quanzhou Women's and Children's Hospital, Quanzhou, China; ^2^Department of Obstetrics and Gynecology, Key Laboratory for Major Obstetric Diseases of Guangdong Province, The Third Affiliated Hospital of Guangzhou Medical University, Guangzhou, China; ^3^Key Laboratory of Reproduction and Genetics of Guangdong Higher Education Institutes, The Third Affiliated Hospital of Guangzhou Medical University, Guangzhou, China; ^4^Prenatal Diagnosis Center, Quanzhou Women's and Children's Hospital, Quanzhou, China; ^5^Department of Public Health for Women and Children, Quanzhou Women's and Children's Hospital, Quanzhou, China; ^6^Ultrasonography, The Second Affiliated Hospital of Fujian Medical University, Quanzhou, China

**Keywords:** fetal growth restriction, chromosomal microarray analysis, karyotype analysis, prenatal diagnosis, copy number variants (CNVs)

## Abstract

**Background:** An increase in pathogenic copy number variants (pCNVs) has been recognized to associate with fetal growth restriction (FGR). Here, we aim to explore the application value of chromosomal microarray analysis (CMA) in prenatal diagnosis of FGR.

**Methods:** Prenatal ultrasound was applied to identify FGR. A total of 149 pregnant women with FGR were enrolled in our study. All subjects underwent karyotype analysis and CMA to reveal the chromosomal abnormalities.

**Results:** In this study, all subjects were successfully detected by karyotype and CMA analyses. Of these subjects, the chromosomal abnormalities detection rate was 5.37% (8/149) for karyotyping and 13.42% (20/149) for CMA, respectively. Among them, an 8.05% (12/149) incremental yield of CMA over karyotype analysis was observed (p = 0.004). In addition, a significant difference of pCNV detection rate was observed between the groups with different high-risk factors (*p* = 0.005). The FGR with structural anomalies group showed the highest pCNV detection rate (33.33%), followed by the FGR with non-structural anomalies group (8.77%) and the isolated FGR group (8.06%).

**Conclusion:** In conclusion, CMA technology showed an effective application value in etiology diagnosis of FGR. We believe that CMA should be recommended as first-line detection technology for prenatal diagnosis in FGR.

## Background

Fetal growth restriction (FGR) refers to the fetus that has not reached its growth potential, and the weight or abdominal circumference is below the 10^th^ percentile of its gestational age. Currently, FGR is one of the most common and complex diseases in obstetrics, which is an important factor for perinatal morbidity and death. Moreover, it may also result in long-term adverse outcomes, including childhood cognitive impairment and increased adult diseases (1). Studies have shown that chromosomal abnormalities can explain 15 to 20% of FGR (2). Therefore, it is of great value in the early screening and diagnosis of FGR.

Although the karyotype analysis technology can identify the large structural variants and chromosome aneuploidy in FGR, the resolution is still limited. Chromosomal microarray analysis (CMA) showed great advantages over traditional karyotype analysis, including array-based comparative genomic hybridization (aCGH) technology and single-nucleotide polymorphism array (SNP array) technology. Among them, SNP array technology can not only provide information of copy number variants but also identify loss of heterozygosity (LOH), uniparental disomy (UPD), and triploid (3–5). A large-scale study indicated that CMA can detect an additional 10% of pathogenic copy number variants over karyotype analysis in FGR with structural abnormalities (6). Moreover, present studies revealed that LOH and UPD are also observed in FGR (7, 8).

To date, there are few studies or with limited subjects available on the application of CMA in the genetic etiology diagnosis of FGR. Our initial aim was to explore the application value of CMA technology in the prenatal diagnosis of FGR, which was the first large-scale study in Fujian province to our knowledge.

## Materials and Methods

### Subjects

A total of 171 singleton pregnant women from January 2017 to December 2019, who were diagnosed with FGR by fetal ultrasound in our hospital, were enrolled. Among them, 149 cases underwent interventional prenatal diagnosis. In the study, multiple pregnancies, cytomegalovirus infection, and chronic diseases related to drug use and abuse were excluded. All subjects signed an informed consent form and this study obtained approval from the Ethics Committee of Quanzhou Women's and Children's Hospital (2020No.31).

### Diagnostic Standard

Fetal crown rump length (CRL) was measured by ultrasound during the first trimester of pregnancy to assess the gestation. In the second trimester, two-dimensional obstetric ultrasound is used to evaluate the growth indicators of the fetus, and the weight of the fetus is estimated based on the growth status of different gestational weeks. Hadlock formula is utilized to calculate the estimated fetal weight (EFW) from the biparietal diameter, abdominal circumference, and femur length. FGR is diagnosed when EFW is below the 10^th^ percentile of gestational age. Additionally, according to the standard of the International Society of Ultrasound in Obstetrics & Gynecology, the fetal growth curve was drawn to evaluate the growth potential of the fetuses. In general, fetal ultrasound structural anomalies referred to morphological defects in fetal organs or parts of the body, such as cleft lip and palate, spina bifida, etc., while other anomalies without any structural anomalies were defined as non-structural anomalies, such as dilatation of the lateral ventricles, enhanced intestinal echo, etc.

### Karyotype Analysis

A total of 149 pregnant women with FGR received genetic counseling and signed informed consent. Ultrasound-guided amniocentesis was performed at gestational age of 16–24 weeks, and 30 ml of amniotic fluid was drawn. The 20 ml amniotic fluid was analyzed according to the amniotic fluid karyotype operation procedure of the prenatal diagnosis department of our hospital (9).

### SNP Array Analysis

The remaining 10 ml amniotic fluid is used for chromosomal microarray analysis. DNA extractions were performed using the QIAamp DNA Blood Kit (QIAGEN, Hilden, Germany) following the kit Handbook (www.qiagen.com). The detection of SNP array is performed in accordance with the standard experimental procedure of the Affymetrix CytoScan 750 K chip kit. The pathogenicity of copy number variations was interpreted with reference to DGV (http://dgv.tcag.ca/dgv), OMIM (https://omim.org/), DECIPHER (https://decipher.sanger.ac.uk/), PubMed (https://www.ncbi.nlm.nih.gov/pubmed/), and other databases.

### Statistical Analysis

The SPSS20.0 software was used for data analysis. The chi-square test was used for statistical analysis among the groups, and the Fisher exact probability test was used for statistical analysis when the chi-square test is not satisfied. A value of *p* < 0.05 was considered as statistical significance.

## Results

### Cases Information

A total of 149 subjects underwent interventional amniocentesis prenatal diagnosis, with maternal age range of 20–46 years and gestational age range of 16–24 weeks. In the present study, all cases were divided into three groups, including the FGR with ultrasound structural anomalies group (*n* = 30), FGR with non-structural anomalies group (*n* = 57), and isolated FGR group (*n* = 62).

### Chromosomal Abnormalities Detected by Karyotyping

In this study, all subjects were successfully detected by the karyotyping and CMA. Among them, three cases of chromosome aneuploidy and five cases of chromosomal structural abnormalities were detected by karyotype analysis (Table 1), with a chromosomal abnormalities detection rate being 5.37% (8/149). In addition, one case of small supernumerary marker chromosome and two cases of chromosomal polymorphism were also detected.

**Table 1:** Comparison of chromosomal abnormalities detected by karyotyping and CMA.

**Karyotype**	**CMA**	**Ultrasound anomalies associated with FGR**	**Inheritance**	**Pregnancy outcome**
T21	arr(21)x3,pCNVs	Isolated	/	TOP
T18	arr(18)x3,pCNVs	Structural	/	TOP
45,X	arr(X)x1,pCNVs	Isolated	/	TOP
46,XN,der(8)t(8;22)(p21.1;q13.1)	arr[hg19]8p23.3p21.1(158,048-28,689,154)x1, 18p11.31p11.23(6,723,060-7,824,813)x3, 22q13.1q13.33(40,195,209-51,197,838)x3,pCNVs	Structural	*De novo*	Stillborn
46,XN,der(6)t(4;6)(q28.3;p25.2)	arr[hg19]4q28.3q35.2(131,389,050-190,957,460)x3,6p25.3p25.2(376,722-3,552,492)x1,pCNVs	Structural	/	TOP
46,XN,der(22)t(11;22)(q23.3;q11.2)	arr[hg19]7p12.1(51,690,431-53,295,058)x3, 11q23.3q25(116,684,163-134,938,470)x3, 22q11.1q11.21(16,888,899-20,716,903)x3,pCNVs	Structural	/	Stillborn
46,XN,dup(4)(p15?)	arr[hg19]4p16.3p16.2(68,345-5,440,181)x1, 4p16.2p15.1(5,447,464-34,170,864)x3,pCNVs	Structural	/	Stillborn
46,XN,del(5)(p13)	arr[hg19]5p15.33p13.3(113,576-29,437,705)x1,pCNVs	Structural	*De novo*	TOP
47,XN,+mar	arr[hg19]13q33.1q34(103,144,279-115,107,733)x3,pCNVs	Non-structural	/	TOP
46,XN	arr[hg19]16q11.2(46,503,19246,925,074)x3, 17q11.2(29,075,556-30,298,421)x1,pCNVs	Non-structural	*De novo*	TOP
46,XN	arr[hg19]17p13.3(1,323,985-2,825,460)x3,pCNVs	Non-structural	*De novo*	TOP
46,XN	arr[hg19]22q11.21(18,648,855-21,800,471)x1,pCNVs	Structural	/	TOP
46,XN	arr[hg19]7q36.1(151,797,859-152,047,392)x1,pCNVs	Non-structural	Maternal	TOP
46,XN	arr[hg19]14q13.2q21.3(35,428,813-49,221,239)x2hmz,pCNVs	Structural	/	TOP
46,XN	arr[hg19]Xp22.31(6,455,151-8,145,527)x1,pCNVs	Isolated	/	Stillborn
46,XN	arr[hg19]Xp22.31(6,455,151-8,141,076)x0, Yq11.222(20,618,887-21,028,944)x2,pCNVs	Isolated	/	Born
46,XN	arr[hg19]Xq28(154,109,413-154,983,124)x3,pCNVs	Structural	/	Born
46,XN	arr[hg19]Yq11.23(26,527,669-27,448,831)x0,pCNVs	Isolated	Paternal	Born
46,XN	arr[hg19]9p24.3p24.1(208,454-8,927,516)x1,pCNVs	Structural	/	TOP
46,XN	arr[hg19]2q12.3q13(109,143,782-110,492,659)x1,pCNVs	Non-structural	/	TOP

*TOP, termination of pregnancy*.

### Comparison of Chromosomal Abnormalities Detected by Karyotyping and CMA

**Figure 1:**
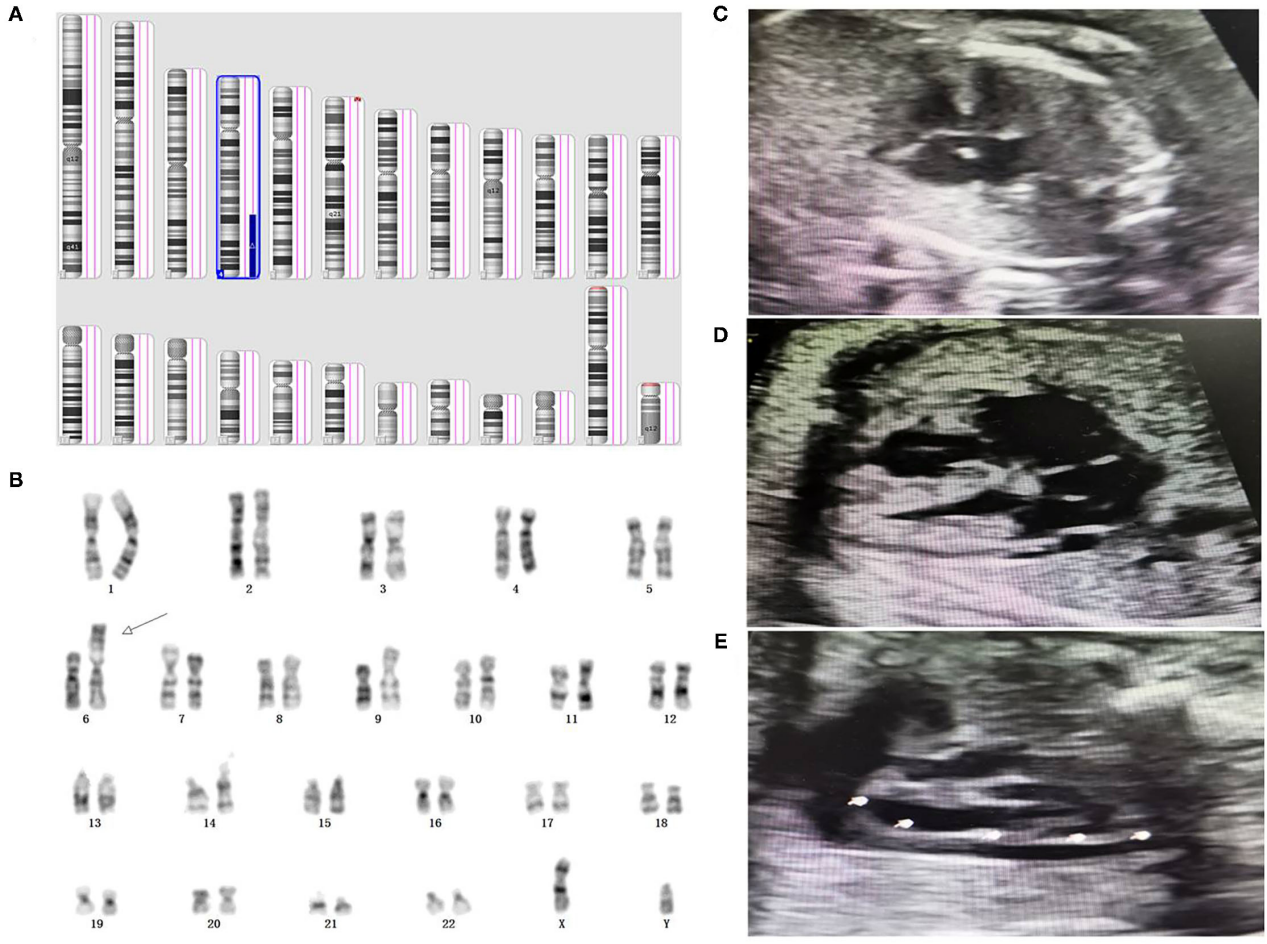
Genetic diagnosis and ultrasound phenotype of derivative chromosome 6. **(A)** SNP array result indicated a 4q28.3q35.2 duplication associated with 6p25.3p25.2 deletion in the fetus. **(B)** Karyotype analysis indicated a derivative chromosome 6 in the fetus. **(C–E)** prenatal ultrasound detection indicated atrioventricular septal defect, coronary sinus dilation, and persistent left superior vena cava in the fetus, respectively.

**Figure 2:**
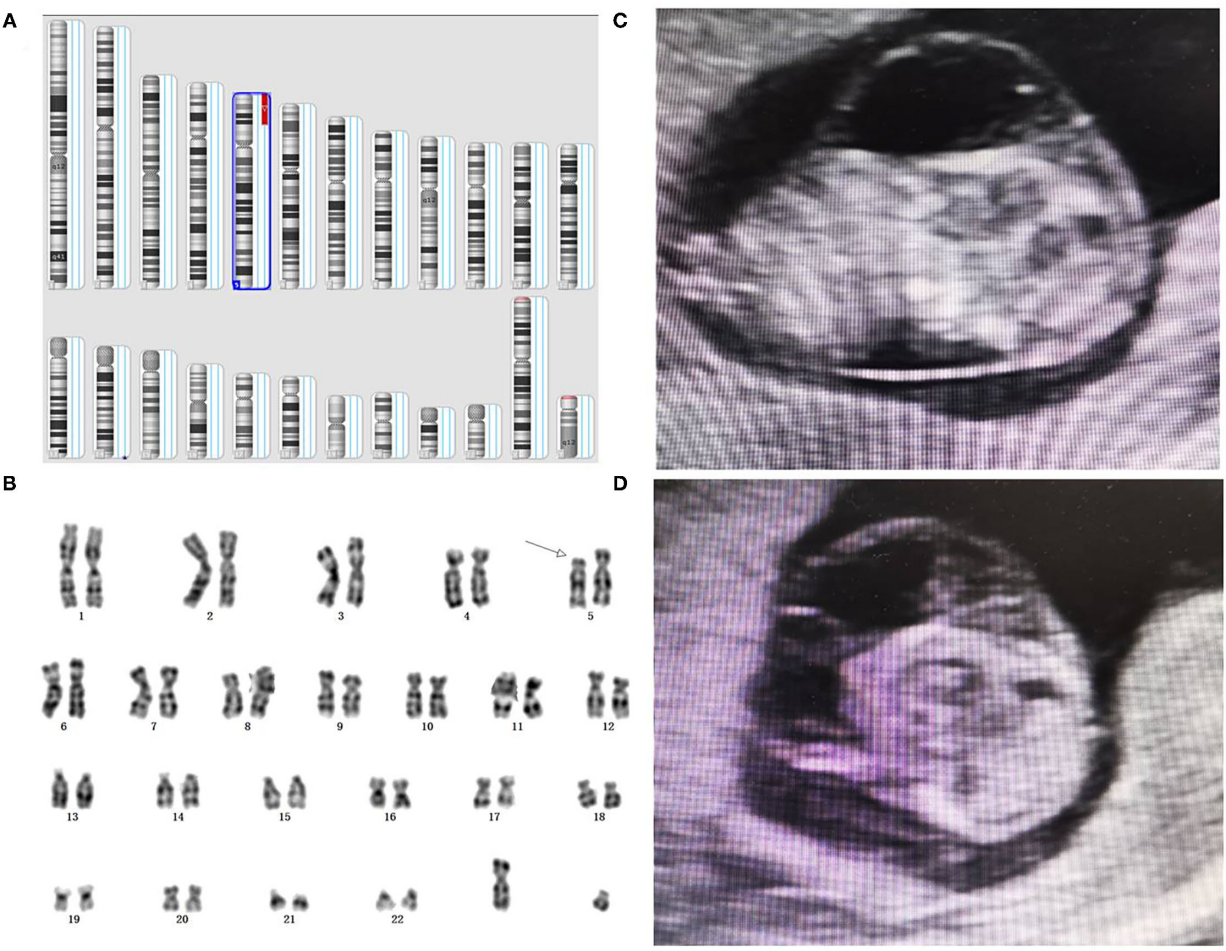
Genetic diagnosis and ultrasound phenotype of partial 5p deletion. **(A)** A detailed view from an SNP array analysis report for chromosomes involved. **(B)** Karyotype analysis revealed a partial 5p deletion in the fetus. **(C, D)** Prenatal ultrasound elicited cystic hygroma colli in the fetus.

In the study, 20 cases of pCNVs were detected by CMA, with a pCNV detection rate of 13.42% (20/149) (Table 1). Twenty cases of variants of unknown significance (VOUS) and one case of benign CNVs (bCNVs) were detected as well (Table 2). Among them, all chromosomal abnormalities detected by karyotyping were confirmed by CMA (Figures 1, 2); moreover, an 8.05% (12/149) incremental yield of CMA over karyotyping was observed (χ^2^ = 8.100, *p* = 0.004) (Table 2). Among them, three cases were from the isolated FGR group (4.84%, 3/62), five cases were from the FGR with non-structural anomalies group (8.77%, 5/57), and four cases were from the FGR with structural anomalies group (13.33%, 4/30). Additionally, one case of small supernumerary marker chromosome was detected by chromosome karyotype analysis and further confirmed by CMA, which was a pathogenic variant and derived from chromosome 13 (13q33.1q34 microduplication).

**Table 2:** The variants of unknown significance detected by CMA.

**Karyotype**	**CMA**	**Ultrasound anomalies associated with FGR**	**Inheritance**	**Pregnancy outcome**
46,XN	arr[hg19]Xp22.31(6,538,033-7,072,640)x3,VOUS	Non-structural	/	Born
46,XN,9qh+	arr[hg19]11q22.1(97,744,329-100,023,601)x1,VOUS	Structural	/	Born
46,XN,9qh+	arr[hg19]5p14.3(21,758,499-22,296,824)x1,VOUS	Non-structural	/	Born
46,XN	arr[hg19]10q11.22q11.23(46,252,072-51,817,663)x3,VOUS	Structural	/	TOP
46,XN	arr[hg19]Xp22.33(535,219-738,276)x3 or Yp11.32 (485,219-688,276)x3,VOUS	Structural	/	TOP
46,XN	arr[hg19]11p15.5(372,355-611,609)x1,VOUS	Non-structural	Maternal	Born
46,XN	arr[hg19] 11p15.5(461,372-677,638)x1,VOUS	Non-structural	Maternal	Born
46,XN	arr[hg19]16p13.12p13.11(14,780,640-16,538,596)x1,VOUS	Non-structural	/	TOP
46,XN	arr[hg19]17p13.1p12(7,549,150-11,438,030)x3,VOUS	Isolated	/	Stillborn
46,XN	arr[hg19] 19p13.2(8,096,719-8,497,269)x3,VOUS	Non-structural	/	TOP
46,XN	arr[hg19]4q35.2(187,900,881-188,943,890)x3,VOUS	Structural	/	Born^a^
46,XN	arr[hg19]4q35.2(187,929,331-188,943,890)x3,VOUS	Structural	/	Born
46,XN	arr[hg19]6p25.3p24.1(203,877-12,217,263)x2 hmz, 6p12.3p11.1(47,247,899-58,726,706)x2 hmz, 6q16.3q24.2(105,137,251-144,038,185)x2 hmz, VOUS	Non-structural	/	Stillborn
46,XN	arr[hg19]6p22.3p21.31(24,654,265-35,934,695)x2hmz,VOUS	Structural	/	Stillborn
46,XN	arr[hg19]Xp22.33(385,561-1,234,634)x3 or Yp11.32 (335,561-1,184,634)x3,VOUS	Non-structural	/	Born
46,XN	arr[hg19]1q43(237,843,614-240,867,477)x3,VOUS	Isolated	/	Born
46,XN	arr[hg19]2p12(78,631,709-79,851,089)x4,VOUS	Isolated	Paternal	Born
46,XN	arr[hg19]4p15.2(21,563,614-22,631,787)x3,VOUS	Isolated	Paternal	Stillborn
46,XN	arr[hg19]Xq28(152,171,288-152,582,007)x1,VOUS	Structural	/	Born^b^
46,XN	arr[hg19]Xq27.1(138,553,702-139,314,460)x2,VOUS	Non-structural	Maternal	TOP

*TOP, termination of pregnancy*;

a *Born with kidney duplication and hydronephrosis in the left kidney*;

b *Language and motor developmental delay occurred after birth*.

### Comparison of the Detection Rates of CNVs Among the Groups

In this study, we further analyzed the detection rate of pCNVs in groups of FGR with different ultrasound phenotypes (Table 3). A significant difference of pCNV detection rates was observed among the groups (χ^2^ = 10.678, *p* = 0.005). Among them, the FGR with structural anomalies group showed a higher pCNV detection rate than that in the FGR with non-structural anomalies group and isolated FGR group (χ^2^ = 8.310, *p* = 0.004; χ^2^ = 9.460, *p* = 0.002). In addition, no significant difference of pCNV detection rates was observed between the FGR with non-structural anomalies group and isolated FGR group (χ^2^ = 0.019, *p* = 0.889).

**Table 3:** Detection rates of CNVs among FGR groups with different ultrasound anomalies.

**Groups**	**Cases**	**pCNVs**	**VOUS**	**bCNVs**	**pCNV detection rates**
FGR with structure anomalies	30	10	7	0	33.33% (10/30)
FGR with non-structural anomalies	57	5	9	0	8.77% (5/57)
Isolated FGR	62	5	4	1	8.06% (5/62)

### Follow-Ups of Pregnancy Outcome

In this study, 90.60% (135/149) were successfully followed up. Among the cases with pCNVs, 13 of them chose to terminate their pregnancy, four fetuses died *in utero*, and three cases continued their pregnancy without obvious abnormality observed after birth (Table 1). Of the cases with VOUS, five cases chose to terminate their pregnancy (two cases with structural anomalies and three cases with soft index anomalies), four cases encountered intrauterine death (three cases with structural anomalies and 1 cases was isolated FGR), and 11 cases continued their pregnancy. The follow-up results showed that one case (4q35.2 microduplication) exhibited kidney duplication and hydronephrosis in the left kidney, and one case (Xq28 microdeletion) exhibited language and motor developmental delay. No obvious abnormalities were observed in the remaining cases.

## Discussion

CMA technology can detect unbalanced CNVs effectively, which has been recommended as a first-line detection technology for the genetic etiology diagnosis in fetus with structural anomalies (10). However, there are few studies available in the application of CMA for FGR genetic etiology diagnosis. Research on the application of CMA technology in FGR with normal karyotypes showed a 10% incremental yield of CMA over karyotyping in FGR with structural anomalies; and 4% pathogenic copy number variation can also be detected in FGR with non-structural anomalies (6). In our study, 12 cases with pCNVs were detected by CMA, with an additional detection rate of 8.05% (12/149) over karyotyping. This study is similar to that reported in the literature (2, 6, 11, 12), which showed a great advantage of CMA technology in the prenatal diagnosis of FGR over karyotyping.

In the present study, based on the ultrasound phenotypes, three groups were divided; a significant difference of pCNV detection rate was observed among the groups. Among them, the detection rate of pCNVs in the FGR with structural anomalies group showed higher pCNV detection rate. This further strengthens the application value of CMA in fetuses with structural anomalies. Moreover, we believe that FGR combined with ultrasound soft indicator anomalies may not increase the risk of chromosomal abnormality.

Chromosome aneuploidy can explain approximately 7% of FGR, of which trisomy 18 was the most common etiologic factor (13). Additionally, studies have shown that 90% of trisomy 18 can express FGR, while it exists in 30% of trisomy 21 (14). Three cases of chromosome aneuploidy were identified in this study, including one case of trisomy 21, one case of trisomy 18, and one case of Turner syndrome. Among them, two cases exhibited isolated FGR. Thus, the genetic diagnosis and clinical consultation of isolated FGR should also be given equal attention. In addition, five cases of common pCNVs associated with FGR were detected (6), including two cases of Xp22.31 microdeletion, one case of 22q11.21 microdeletion syndrome, one case of 22q11.1q11.21 microduplication syndrome, and one case 4p16.3 microdeletion syndrome. Previous studies have shown that Xp22.31 microdeletion in fetuses only exhibited isolated FGR (12, 15). In this study, two cases of Xp22.31 microdeletion were detected with isolated FGR, which is consistent with the previous reports. Studies have shown that 22q11.21 microdeletion was detected in FGR with structural malformations (16). In this study, a 22q11.21 microdeletion in FGR without cardiac abnormality was observed, which may relate to the non-penetrance in 22q11.21 microdeletion. Moreover, researchers indicated that the 22q11.21 microduplication can also be observed in FGR with oligohydramnios or isolated FGR (6, 17). The 4p16.3 microdeletion covering the “Wolf–Hirschhorn syndrome” region would exhibit intellectual disability, developmental delay, and hypotonia, and has been identified in FGR previously (16, 18). In this study, the fetus with 4p16.3 microdeletion referred to as Wolf–Hirschhorn syndrome exhibited FGR with multiple structural anomalies.

VOUS is a great challenge in clinical consultation; because of low ratio of parent verification and the incomplete localized database, it is difficult to interpret the pathogenicity of the subjects with VOUS. Thus, it is very important to apply fetal ultrasound to further monitor the growth and development of the fetuses. In the current study, 20 cases of VOUS were detected in this study, which may be the etiology for FGR. Among them, one case of Xq28 region involving 410.7kb deletion was detected, including *PNMA3, PNMA6A*, and *MAGEA1* genes. As shown in the database, there are cases with similar and smaller deletions than the fragment exhibiting developmental delay and ventricular septal defect. Moreover, a previous study showed that a patient who harbored a deletion of chromosome Xq28 exhibited growth delay (19). Ultrasound in this case showed ventricular septal defect and FGR, which was similar to the clinical phenotypes reported in the database and literature. In addition, an unknown significance of Xp22.33 microduplication was detected, containing *SHOX/SHOXY* gene, which was related to autosomal dominant diseases of Leri–Weill cartilage osteogenesis disorder due to gene mutation or deletion. Previous studies reported that duplication involving the *SHOX* gene would lead to short stature, intellectual disability, and developmental delay (20, 21). Thus, in the present study, we believe that Xq28 deletion and Xp22.33 duplication may be the genetic etiology for FGR. It can be seen that prenatal ultrasound combined with CMA detection technology would be helpful for the clinical consultation of VOUS and the exploration of genotype–phenotype relationship.

To date, more studies have shown that UPD will result in FGR (22–24). Our study identified a loss of heterozygosity in the 14q13.2q21.3 region who exhibit FGR combined with ventricular septal defect, auricle abnormality and hypoxia. Studies have shown that maternal UPD of chromosome 14 will result in Temple syndrome, which have been reported to be associate with short stature, intrauterine growth retardation, abnormal facial appearance, and low birth weight (25), while paternal UPD of chromosome 14 will leading to Kagami-Ogata syndrome, with the clinical phenotypes including abnormal bone development, joint contractures, abnormal facial features, developmental delay, intellectual disability, etc (26, 27). According to the phenotype, the maternal 14q13.2q21.3 loss of heterozygosity may harbor. However, more work should be done to determine the source of loss of heterozygosity in this case.

## Conclusions

In conclusion, the genetic etiology of FGR is complicated, including chromosome aneuploidy, UPD, LOH, and copy number variants. In this study, a 4.84% incremental yield of CMA over karyotyping was observed in isolated FGR. Thus, we believe that CMA should be recommended as a first-line detection technology for prenatal diagnosis in FGR, in the presence of other ultrasound anomalies or not. Moreover, our research indicated that Xp28 microdeletion and Xp22.33 duplication may be the genetic etiology for FGR.

## Data Availability Statement

The raw data supporting the conclusions of this article will be made available by the authors, without undue reservation.

## Ethics Statement

The studies involving human participants were reviewed and approved by the Institutional Ethics Committee of Quanzhou women's and children's hospital (2020No.31). The patients/participants provided their written informed consent to participate in this study. Written informed consent was obtained from the individual(s) for the publication of any potentially identifiable images or data included in this article.

## Author Contributions

GL designed the study. YC and JZ wrote the article. JZ, QL, and SZ performed routine chromosome analysis, SNP array detection, and data analysis. GL, LS, YX, and YJ modified and proofread the paper. All authors approved the final article.

## Funding

This research was supported by the Fujian Provincial Health Commission Youth Science and Technology Project (2020QNB045); Quanzhou Science and Technology Bureau (2020C026R).

## Conflict of Interest

The authors declare that the research was conducted in the absence of any commercial or financial relationships that could be construed as a potential conflict of interest.

## Publisher's Note

All claims expressed in this article are solely those of the authors and do not necessarily represent those of their affiliated organizations, or those of the publisher, the editors and the reviewers. Any product that may be evaluated in this article, or claim that may be made by its manufacturer, is not guaranteed or endorsed by the publisher.
